# Determinants of lost-to-follow-up (LTFU) among National Health Insurance Scheme-insured hypertension and diabetes patients attending accredited health facilities in Ghana

**DOI:** 10.1186/s41182-025-00743-3

**Published:** 2025-05-06

**Authors:** Edward Nketiah-Amponsah, Solomon Ahimah-Agyakwah, Robert Kaba Alhassan, Gifty Sunkwa-Mills, G. P. Gómez-Pérez, Judith van Andel, Alex Yao Israel Attachey, Yaw Nyarko Opoku-Boateng, Vivian Addo-Cobbiah, Bernard Okoe-Boye, Tobias Floris Rinke de Wit, Maxwell Akwasi Antwi

**Affiliations:** 1 Department of Economics, University of Ghana, Legon, Accra, Ghana; 2 Centre for Health Policy and Implementation Research, Institute of Health Research, University of Health and Allied Sciences, Ho, Ghana; 3 PharmAccess Foundation, Accra, Ghana; 4 PharmAccess Foundation, Amsterdam, The Netherlands; 5 National Health Insurance Authority, Accra, Ghana; 6 Department of Global Health, University of Amsterdam, Amsterdam, The Netherlands; 7 Amsterdam Institute for Global Health & Development, Amsterdam, The Netherlands

**Keywords:** Hypertension, Diabetes, Lost-to-follow-up, Logistic regression, Ghana National Health Insurance Authority, National Health Insurance Scheme

## Abstract

**Background:**

Hypertension (HPT) and diabetes mellitus (DM) are major contributors to morbidity and mortality in Ghana. A key challenge in managing these conditions is non-adherence to follow-up visits, commonly referred to as "lost- to- follow-up" (LTFU). Data from the National Health Insurance Authority (NHIA) between 2017 and 2019 revealed that 37% (232,442/634,981) of patients were LTFU at NHIA-accredited health facilities. This study aimed to investigate the factors driving this high LTFU rate in Ghana.

**Methods:**

A total of 480 hypertensive and diabetic patients, randomly selected from the NHIA electronic claims database from facilities in the Greater Accra and Ashanti regions between 2019 and 2020, were interviewed. Participants were divided into two groups: LTFU, which consisted of only one visit (351, 73%), and follow-up (FU), which consisted of more than one visit (129, 27%). The sample included patients diagnosed with hypertension only (308, 64%), diabetes only (45, 9%), and both hypertension and diabetes (127, 26%).

**Results:**

No statistically significant socioeconomic differences were observed between the LTFU and FU groups, except in their adherence to follow-up visits. The likelihood of LTFU was higher among patients without follow-up awareness (OR = 2.5, 95% CI: 1.05–4.83), those who felt stigmatized (OR = 15.51, 95% CI: 1.01–238.90), those who attended facilities where physicians were available only some of the time (OR = 7.37, 95% CI: 1.07–50.61), those attending facilities without the necessary diagnostic equipment, those who described the NHIS coverage for DM diagnostic tests as inadequate, and those receiving traditional or herbal treatments (OR = 16.90, 95% CI: 3.12–91.45). Conversely, patients from the Ashanti Region (OR = 0.58, 95% CI: 0.35–0.96), those educated on diagnostic procedures (OR = 0.28, 95% CI: 0.08–0.98), and those whose treatment was not under control (OR = 0.04, 95% CI: 0.00–0.69) were less likely to be LTFU. Additionally, patients diagnosed more than ten years ago (OR = 0.44, 95% CI: 0.24–0.79) and those who were neutral about establishing support groups were less likely to be LTFU.

**Conclusions:**

The study found that lack of follow-up awareness, stigmatization, and preference for traditional or herbal treatments are key drivers of lost-to-follow-up behavior among hypertension and diabetes patients. Thus, remedial policies should include increasing patient education on the importance of follow-up visits, ensuring the availability of essential medications, diagnostic equipment, and physicians, expanding the NHIA financial coverage, and integrating traditional medicine into standard healthcare to improve treatment adherence and reduce LTFU rates.

**Supplementary Information:**

The online version contains supplementary material available at 10.1186/s41182-025-00743-3.

## Background

Non-communicable diseases (NCDs) such as cancers, hypertension (HPT), and diabetes mellitus (DM) are a growing public health concern in Sub-Saharan Africa (SSA). In Ghana, NCDs are a major cause of morbidity and mortality [1, 2] and account for 43% of deaths in the country [3]. The prevalence of hypertension is approximately 13%, while diabetes affects between 6.2 and 13.9% of adults aged 15–49 years in Ghana [4]. Furthermore, an estimated 66% of individuals with hypertension and 60% of those with diabetes in Africa, including Ghana, remain undiagnosed [5, 6], putting them at heightened risk of developing preventable complications, such as stroke and heart attack, and experiencing premature death [5]. Among those diagnosed, only 31% of hypertension patients in Africa receive medical treatment, with just 6.5% achieving disease control [5, 7].

The growing burden of hypertension and diabetes in Ghana threatens the country’s ability to achieve Sustainable Development Goal (SDG) 3, particularly target 3.4, which aims to reduce premature deaths from NCDs by one-third by 2030 through prevention and treatment [8]. Ghana’s healthcare system currently faces challenges in managing both infectious and non-infectious diseases, highlighting the need for strategic interventions to address this double burden [7, 9]. Without effective action, Ghana risks failing to achieve universal health coverage, as envisioned by SDG target 3.8, by 2030 [1, 5, 10].

The economic implications of the increasing non-communicable disease (NCD) burden are evident, with NCDs contributing to a loss of between 1 and 5% of Ghana’s Gross Domestic Product (GDP) [11, 12]. At the household level, conditions like hypertension and diabetes exacerbate financial risks due to their impact on economic productivity and the financial burden of long-term treatment [13].

**Fig. 1:**
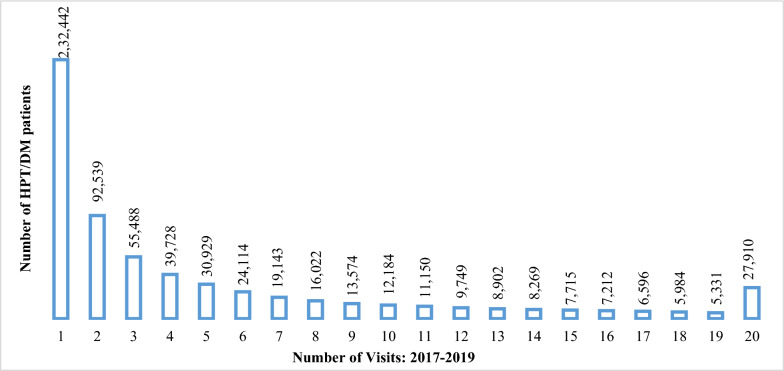
Hypertension/diabetes patients by number of visits: 2017–2019. Source: PharmAccess & NHIA Analytic Data (2021)

One major challenge in managing hypertension and diabetes in Ghana is patient non-adherence to physician-recommended follow-up visits and treatment. Analysis of the NHIA electronic claims database from 2017 to 2019 revealed a sharp increase in hypertension and diabetes diagnoses among individuals aged 18 years and above. These two NCDs accounted for 14.6% of total electronic claims costs and 4.4% of total NHIS claims nationwide. Most notably, 37% (232,442/634,981) of patients diagnosed with hypertension and diabetes did not return for follow-up visits to the same NHIS service providers for treatment of their diagnoses (Fig. 1).

The proportion of lost-to-follow-up (LTFU) visits among hypertension and diabetes patients in Ghana is approximately 8.4 times higher than the international average of 4.4% for non-communicable disease (NCD) patients, as observed in the Prospective Urban and Rural Epidemiology (PURE) study, which covered 18 countries across different income levels [10]. This is concerning given that only 25–33% of adults with hypertension in Ghana are aware of their condition, and just 80% of men and 63% of women know their status [5]. Moreover, among the few patients receiving treatment for hypertension, only 1.7 to 12.7% have the disease under control [5]. Patients who are lost to follow-up are at increased risk of preventable complications and premature death [3].

Several factors have been identified in the literature as contributing to LTFU, including the cost of treatment and issues with health insurance [14–16], negative side effects of medications [17], the timing of diagnosis and severity of disease [15], and difficulty in accessing healthcare due to distance from health facilities [16–18]. Poor treatment outcomes [19], preferences for alternative treatments such as traditional or homemade remedies [20, 21], limited understanding of NCD management, and a lack of awareness about follow-up [21, 22] have also been cited as important determinants of LTFU behavior. Provider-specific factors, including poor service quality, long waiting times, unfriendly or disrespectful behavior from healthcare staff, and lack of medical equipment, have further been linked to LTFU [22–24]. Additionally, demographic factors such as age, gender, educational attainment, and race have been reported to contribute to LTFU [25–29].

However, the specific drivers of LTFU among hypertensive and diabetic patients insured by the NHIS and utilizing credentialed health facilities in Ghana remain unexplored. This study hypothesizes that socio-demographic, patient-related, provider-related, treatment-related, and financial factors do not influence LTFU behavior. The findings from this study aim to fill this gap in the literature and inform policies and practices to improve the treatment, management, and control of NCDs in Ghana.

## Methods

### Study setting

The study was conducted in the Greater Accra and Ashanti regions of Ghana. These regions were selected because they have the largest number of NHIS-insured members and account for most electronic claims submitted by credentialed health facilities. Additionally, they have the highest prevalence, morbidity, and mortality rates for hypertension and diabetes [7]. Moreover, the two regions are more urbanized and have residents with diverse socio-demographic characteristics, including educational attainment and income levels [3, 30]. For instance, between 1973 and 2009, the prevalence of hypertension in urban areas of Ghana was 54.6%, compared to about 19.3% in rural areas [30].

### Study design and sampling

This study employed a quantitative, cross-sectional design using structured questionnaires. The questionnaire developed by the authors was an adaptation of the World Health Organization’s STEPS Instrument [29], modified to capture additional socio-demographic data. Data on healthcare utilization and patient visits were extracted from the NHIA electronic claims database. The sample size was determined using the OpenEpi Version 3 open-source calculator SSPropor.^1^,^2^Based on response rates from prior non-communicable disease (NCD) studies in Ghana [3, 30], the study hypothesized a frequency outcome factor of at least 80%, with confidence limits of ± 5%. The calculation, with a design effect of 2, yielded 492 patients and was subsequently increased to 510 patients to account for non-response. Respondents with diagnoses of hypertension, diabetes, or both were selected from NHIS-accredited health facilities in the Greater Accra and Ashanti regions using simple random sampling without replacement. Respondents were categorized into two groups: LTFU (lost- to- follow-up) and FU (follow-up). The LTFU group consisted of patients who visited a health facility only once in 2019 for hypertension and diabetes treatment or management and did not return for any follow-up in 2020. The FU group consisted of patients who had multiple visits (2 or more) in 2019 and at least one follow-up visit in 2020 for hypertension, diabetes, or any other illness. Although the NHIA database used for the study comprised patients with clinically diagnosed hypertension and diabetes, sampled respondents were asked to confirm their status as being hypertensive, diabetic, or both before the interview. Those with incomplete, missing, or inaccurate data (e.g., incorrect contact information or denial of hypertension and diabetes diagnoses) were excluded. Questionnaires were administered face-to-face at respondents’ homes, offices, or other agreed-upon locations. Telephone interviews were conducted with respondents who were unable to attend face-to-face interviews.

### Data collection

Six field assistants (FAs) with backgrounds in public health were involved in data collection under the supervision of a health economist. The FAs received two rounds of training. The first was a three-day classroom training on hypertension, diabetes, and other NCDs, covering their causes, symptoms, diagnoses, and standard treatment protocols, as well as NHIA claims verification procedures and patient LTFU behavior. The second phase involved field training, during which each FA interviewed at least three respondents to pre-test the questionnaire and ensure a thorough understanding of the data collection process. The field data were collected using the Computer-Assisted Personal Interviewing (CAPI) mobile application. Overall, 480 respondents were interviewed.

### Ethical considerations

Informed consent was obtained from all participants before their inclusion in the study. Respondents were provided with information about the purpose, venue, duration, and topics to be discussed prior to the interviews. Written informed consent was also obtained before administering the questionnaire. Respondents who chose to discontinue the interview due to the sensitivity of questions or personal reasons were allowed to withdraw. Ethical clearance for the study was granted by the University of Ghana’s Institute of Statistical, Social, and Economic Research (ISSER) Review Board’s Ethics Committee for Humanities, under clearance number ECH 265/21-22. It is also instructive to note that approval was granted by the NHIA for the use of the electronic claims data for the purposes of sampling our respondents. The NHIA is also a collaborator in the study.

### Data analysis

The collected data were categorized into five main determinants of LTFU: respondents’ socio-demographic characteristics, disease characteristics (hypertension and/or diabetes), provider characteristics, treatment issues, and financial/NHIA issues (see Tables 2, 3, 4). Each category of determinants was quantitatively analyzed using multivariate logistic regression with Stata version 15.1 [31]. The analysis aimed to include as many determinants of LTFU behavior as possible without leading to multicollinearity, which can arise when several variables are included in a single model. To mitigate this, variables were grouped into vectors under each category of determinants, as using a single model for all variables would result in over-parameterization. The dependent or outcome variable for the empirical analysis is ‘lost to follow-up’ (LTFU), which is conceptualized and defined in this study as a patient who made a single visit to an accredited NHIS facility in 2019 for the treatment or management of hypertension and diabetes but did not make any further visit in 2020. Consequently, the variable is coded 1 if the patient made only one visit in 2019 for hypertension and/or diabetes care and no further visit in 2020 and 0 otherwise. Our key explanatory variables encompass patients’ awareness of the need for follow-up visits for chronic health conditions (hypertension and diabetes); whether a patient felt or perceived herself as stigmatized by healthcare workers due to being discriminated against, insulted, or disrespected on account of her health condition; and whether the patient uses orthodox medication or herbal/traditional remedies for treatment. The definitions and measurements of the key variables used for the various estimations are detailed in Appendix Table 1.2. Since this is a case–control study, almost all the variables used for the analysis are either binary or categorical and not scaled with ordinal ratings. However, a reliability test on selected categorical variables revealed relatively high validity and reliability, with Cronbach's alpha of 0.7 or higher. Following the empirical approach in the literature [32–35], multivariate logistic regression models (with adjusted odds ratios) were estimated for the different categories of determinants.

## Results

### Data description

A total of 480 respondents were interviewed, comprising 351 (73%) lost-to-follow-up (LTFU) patients and 129 (27%) follow-up (FU) patients (Table 1). The sample included individuals diagnosed with hypertension (HPT) only (308, 63%), diabetes (DM) only (45, 9%), and both hypertension and diabetes (127, 26%), with participants from the Greater Accra (234, 49%) and Ashanti (246, 51%) regions. The majority of respondents were female (299, 62%) compared to males (181, 38%), and most had completed Junior High School or lower education levels (385, 80%).

**Table 1:** Socio-demographic characteristics of respondents by patient group

Patient type	LTFU group		FU group		Total		Pearson *χ*^2^	
	*n*	%	*n*	%	*N*	%	Stat.	Prob.
	351	73%	129	27%	480	100%		
By region								
Greater Accra	178	51%	56	43%	234	49%	2.01	0.16
Ashanti	173	49%	73	57%	246	51%		
By HPT/DM prevalence								
HPT or DM only	260	74%	93	72%	353	74%	0.19	0.66
HPT&DM	91	26%	36	28%	127	26%		
By gender								
Male	136	39%	45	35%	181	38%	0.60	0.44
Female	215	61%	84	65%	299	62%		
By educational level								
Junior secondary or below completed	284	81%	101	78%	385	80%	0.41	0.52
High school or above completed	67	19%	28	22%	95	20%		
By age								
Adults (aged 25–65)	274	78%	95	74%	369	77%	1.04	0.31
Seniors (aged above 65)	77	22%	34	26%	111	23%		
By ethnicity								
Akan	232	66%	88	68%	320	67%	0.19	0.66
Non-Akan	119	34%	41	32%	160	33%		
By marital status								
Single	119	34%	41	32%	160	33%	0.19	0.66
Married	232	66%	88	68%	320	67%		
By work status								
Employed	257	73%	90	70%	347	72%	0.56	0.45
Not employed	94	27%	39	30%	133	28%		
By household size								
Below national mean (< 3.9)	105	30%	38	29%	143	30%	0.11	0.74
Above national mean (≥ 4)	246	70%	91	71%	337	70%		
By annual income (GH₵)								
Below national mean (< 11,692)	236	67%	85	66%	321	67%	0.08	0.78
Above national mean (≥ 11,692)	115	33%	44	34%	159	33%		

The figures for mean household size and mean income per-capita are based on the Ghana Statistical Service’s Ghana Living Standards Survey Round 7. Data source: Field Data (2022)

The age distribution showed that 369 respondents (77%) were adults, while the ethnic characteristics showed the regional demographics, with Akan (320, 67%) being the predominant group, followed by Ga-Dangme (69, 14%), Ewe (46, 10%), and other ethnicities (45, 9%). For marital and employment status, there were more married respondents (67%) than unmarried respondents, and more employed respondents (72%) than unemployed respondents. Additionally, most respondents (337, 70%) lived in households with four or more members, which exceeds the national average household size. The majority (321, 67%) had annual income below the national per-capita income of GH₵ 11,692, as reported in [36].

A Pearson’s chi-square test (*χ*^2^) revealed no statistically significant differences in the socio-demographic characteristics between the LTFU and FU groups, suggesting comparability between them, with the main difference being their adherence to follow-up visits. This aligns with findings in related studies [37, 38].

### Lost to follow-up (LTFU) results

The odds ratios (OR) for each determinant of LTFU are presented in Tables 2, 3, 4. An OR greater than 1 indicates a higher likelihood of LTFU, an OR equal to 1 indicates the same likelihood, while an OR less than 1 indicates a lower likelihood. Pre-estimation tests for multicollinearity, using the variance inflation factor (VIF), revealed no severe multicollinearity, with mean VIF values ranging from 1.09 to 4.44, which is below the threshold of 10 [34].

**Table 2:** Socio-demographic determinants of LTFU behavior

Dependent variable: HPT/DM LTFU group (= 1)	Odds ratio [95% CI]
Sex	
Male	Ref.
Female	0.78 [0.49–1.26]
Region	
Greater Accra	Ref.
Ashanti	0.58 [0.35–0.96]**
Age	
Adults (25–64 years)	Ref.
Seniors (≥ 65 years)	0.87 [0.53–1.43]
Household size	
Small size (≤ 3)	Ref.
Medium-size (4–6)	1.20 [0.69–2.11]
Large size (≥ 7)	0.58 [0.31–1.11]*
Education level	
≤ Primary	Ref.
≥ Secondary	0.66 [0.42–1.03]*
Ethnicity	
Akan	Ref.
Non-Akan	0.79 [0.46–1.36]
Marital status	
Single	Ref.
Married	0.95 [0.55–1.64]
Constant	6.26 [2.81–13.93]***
Mean VIF	1.30
Observations	439.00
Log likelihood	− 249.41
Wald *χ*^2^	16.02
Pearson *χ*^2^	0.04
Hosmer–Lemeshow goodness-of-fit test	0.96

Data source: Field Data (2022)

***Significant at 1%; **significant at 5% and *significant at 10%

**Table 3:** Patient's HPT/DM status determinants of LTFU behavior

Dependent variable: HPT/DM LTFU group (= 1)	Odds ratio [95% CI]
Patient's HPT/DM status	
HPT/DM	Ref.
HPT only	0.77 [0.44–1.35]
DM only	0.63 [0.25–1.55]
First diagnostic period	
Diagnostic period (≤ 2 years)	Ref.
Diagnostic period (5–10 years)	0.46 [0.26–0.81]***
Diagnostic period (< 10 years)	0.44 [0.24–0.79]***
Follow-up awareness	
Yes	Ref.
No	2.25 [1.05–4.83]**
Patient currently receiving treatment	
Yes	Ref.
No	1.65 [0.71–3.80]
Family member with NCD	
Yes	Ref.
No	0.85 [0.52–1.39]
Establishing HPT/DM support group	
Strongly recommend	Ref.
Neutral	0.15 [0.03–0.83]**
Constant	5.18 [2.27–9.95]
Mean VIF	1.09
Observations	393.00
Log likelihood	− 214.76
Wald *χ*^2^	18.20
Pearson *χ*^2^	0.02
Hosmer–Lemeshow goodness-of-fit test	0.44

Data source: Field Data (2022)

***Significant at 1%; **Significant at 5% and *significant at 10%

**Table 4:** Provider determinants of LTFU behavior

Dependent variable: HPT/DM LTFU group (= 1)	Odds ratio [95% CI]
Education on HPT/DM diagnostic procedure	
No	Ref.
Yes	0.28 [0.08–0.98] **
Don't remember	0.53 [0.02–17.51]
Existence of follow-up plan	
Yes	Ref.
No	2.62 [0.67–10.24]
Education on condition	
All education	Ref.
Symptoms and control	0.31 [0.08–1.17] *
Causes and control	0.96 [0.10–8.96]
Support group for HPT patients	
Yes	Ref.
No	1.62 [0.33–7.95]
Support group for DM patients	
Yes	Ref.
No	0.61 [0.12–3.04]
Availability of physicians	
Available all the time	Ref.
Available most of the time	3.06 [0.18–51.9]
Available some of the time	7.37 [1.07–50.61] **
HPT diagnostic equipment availability	
Basic equipment available	Ref.
Essential equipment available	0.52 [0.06–4.51]
All required equipment available	0.03 [0.00–0.38] ***
DM diagnostic equipment availability	
All required equipment available	Ref.
Essential equipment available	3.72 [0.39 to 35.22]
Basic equipment available	27.87 [1.74–446.42] **
Availability of HPT medication	
Always available	Ref.
Available most of the time	2.01 [0.22–18.21]
Available some of the time	0.43 [0.03–7.01]
Unavailable always/some of the time	0.16 [0.003–9.03]
Availability of DM medication	
Always available	Ref.
Available most of the time	0.51 [0.05–4.86]
Available some of the time	1.96 [0.12–32.73]
Unavailable always/most of the time	9.21 [0.13–660.06]
Stigmatization of HPT/DM patient	
No stigmatization	Ref.
Stigmatization	15.51 [1.01–238.90]**
Constant	0.12 [0.1–2.83]
Mean VIF	3.33
Observations	93.00
Log likelihood	− 43.90
Wald *χ*^2^	33.03
Pearson *χ*^2^	0.03
Hosmer–Lemeshow goodness-of-fit test	0.21

Data source: Field Data (2022)

***Significant at 1%; **Significant at 5% and *significant at 10%

Post-estimation tests, including the Pearson chi-square and Hosmer–Lemeshow goodness-of-fit tests, confirmed that the models fit the data well and were statistically robust. The *p*-value of the Pearson’s chi-square test for each estimation was below the 5% significance level, indicating that the estimated results were statistically different from random estimation [34]. The Hosmer–Lemeshow goodness-of-fit test also showed no evidence of poor model calibration, even at the 10% significance level [35, 38].

The following section describes the various determinants of LTFU in Tables 2, 3, 4.

### Socio-demographic determinants

Table 2 shows significant regional differences in LTFU behavior, with patients from the Ashanti region (OR = 0.58, 95% CI: 0.35–0.96) being less likely to be LTFU compared to those from the Greater Accra region. Other socio-demographic factors, such as household size and education level, were found to be significant at the 10% level.

### Patient’s diagnosis characteristics

Table 3 reveals that the type of diagnosis (hypertension only, diabetes only, or both) did not significantly influence LTFU behavior. However, the duration since diagnosis had a significant impact. Patients diagnosed 5–10 years prior to the study (OR = 0.46, 95% CI: 0.26–0.81) and those diagnosed more than 10 years ago (OR = 0.44, 95% CI: 0.24–0.79) were less likely to be LTFU than those diagnosed within the last two years. Additionally, patients unaware of the importance of follow-up visits (OR = 2.5, 95% CI: 1.05–4.83) were more likely to be LTFU. Interestingly, patients who were neutral about the establishment of support groups were less likely to be LTFU, a result that contradicted expectations.^3^,^4^

### Provider characteristics

Table 4 also shows that patients who received education on diagnostic procedures at the time of first diagnosis were less likely to be LTFU (OR = 0.28, 95% CI: 0.08–0.98). However, the effect of education on causes, symptoms, and disease control was not significant. The availability of physicians also played a critical role. Patients attending facilities where physicians were only available some of the times were more likely to be LTFU (OR = 7.37, 95% CI: 1.07–50.61). Similarly, the lack of necessary diagnostic equipment increased the likelihood of LTFU, particularly in facilities with only basic equipment for DM (OR = 27.87, 95% CI: 1.74–446.42). Stigmatization at healthcare facilities increased LTFU (OR = 15.51, 95% CI: 1.01–238.90).

### Treatment issues as LTFU determinants

Table 5 highlights that patients receiving non-orthodox treatments, such as traditional (herbal or spiritual) and/or personal/home-made remedies, were much more likely to be LTFU (OR = 16.90, 95% CI: 3.12–91.45) than those receiving only orthodox treatments. Conversely, patients whose treatment was not under control were less likely to be LTFU (OR = 0.04, 95% CI: 0.00–0.69).

**Table 5:** Treatment issues as determinants of LTFU behavior

Dependent variable: HPT/DM LTFU group (= 1)	Odds ratio [95% CI]
Patient's current treatment	
Orthodox only	Ref.
Non-orthodox only	16.90 [3.12–91.45]***
NCD type	
HPT only	Ref.
DM only	0.80 [0.17–3.74]
HPT/DM and others	2.07 [0.59–7.19]
Treatment under control	
Yes	Ref.
No	0.04 [0.00–0.69]**
Poor treatment services	
No	Ref.
Yes	1.05 [0.38–2.88]
Poor treatment outcome	
Yes	1.39 [0.45–4.28]
Prolonged treatment	
No	Ref.
Yes	1.31 [0.28–6.04]
Patient stigmatization	
No	Ref.
Yes	0.23 [0.03–1.77]
Traditional/herbal treatment	
Satisfactory	Ref.
Neutral	0.48 [0.09–2.46]
Unsatisfactory	0.50 [0.14–1.84]
Constant	1.76 [0.76–4.11]
Mean VIF	1.14
Observations	103.00
Log likelihood	− 51.70
Wald *χ*^2^	18.82
Pearson *χ*^2^	0.04
Hosmer–Lemeshow goodness-of-fit test	0.43

Data source: Field Data (2022)

***Significant at 1%; **Significant at 5% and *significant at 10%

### Financial issues as LTFU determinants

As shown in Table 6, inadequate NHIS coverage for DM diagnostic tests increased the likelihood of LTFU (OR = 18.03, 95% CI: 1.45–22.12 for inadequate coverage, OR = 10.41, 95% CI: 4.11–26.34 for very inadequate coverage). Similarly, respondents who recommended extending NHIS medication coverage were more likely to be LTFU (OR = 9.63, 95% CI: 3.45–26.90). On the other hand, patients who found NHIS coverage for DM medication to be very adequate and those who did not recommend the extension of NHIS coverage for HPT medication (OR = 0.00, 95%CI: 0.00–0.00) were less likely to be LTFU (OR = 0.01, 95% CI: 0.00–0.33) (Table 6).

**Table 6:** Financial determinants of LTFU behavior

Dependent variable: HPT/DM LTFU group (= 1)	Odds ratio [95% CI]
NHIS HPT tests coverage	
Adequate	Ref.
Inadequate	0.44 [0.08–2.4]
NHIS HPT medication coverage	
Adequate	Ref.
Inadequate	0.85 [0.08–9.20]
NHIS DM tests coverage	
Very adequate	Ref.
Adequate	1.45 [0.12–17.46]
Inadequate	18.03 [1.45–22.12]**
Very inadequate	10.41 [4.11–26.34]***
NHIS DM medication coverage	
Inadequate	Ref.
Adequate	0.15 [0.02–1.05]*
Very adequate	0.01 [0.001–0.33]***
NHIS HPT tests coverage extension	
Yes	Ref.
No	8.404 [0.58–122.34]
NHIS DM tests coverage extension	
Yes	Ref.
No	1.22 [0.11–11.04]
NHIS HPT medication coverage extension	
Yes	Ref.
No	0.00 [0.00–0.00]***
NHIS DM medication coverage extension	
No	Ref.
Yes	9.63 [3.45–26.90]***
Patient's annual income	
Below median income (GH₵ 5792)	Ref.
Above median income (GH₵ 5792)	3.91 [0.64–23.91]
Patient's out-of-pocket payment	
No	Ref.
Yes	0.99 [0.25–4.02]
Constant	0.52 [0.02–16.13]
Mean VIF	4.44
Observations	64.00
Log likelihood	− 30.42
Wald χ^2^	389.78
Pearson χ^2^	0.00
Hosmer–Lemeshow goodness-of-fit test	0.51

Data source: Field Data (2022)

***Significant at 1%; **Significant at 5% and *significant at 10%

## Discussion

This study investigated various predictors of LTFU behavior among patients diagnosed with hypertension, diabetes or both in the Ashanti and Greater Accra Regions of Ghana. The study findings reveal that the main drivers of lost- to-follow -up among NHIS-insured hypertension and diabetes patients in Ghana are low awareness of the exigency of follow-up visits for chronic diseases; lack of diagnostic equipment at credentialled health facilities, the preference for herbal treatment or traditional medicine over orthodox medicine and perceived stigmatization of patients occasioned by the attitude and conduct of health workers.

The higher participation of female respondents in this study is consistent with prior research showing that women are generally more willing to participate in research studies than men [38–40]. Women also tend to use medical services more frequently, owing to greater morbidity, heightened sensitivity to symptoms, and reproductive health needs [41–44]. However, no gender bias was observed regarding LTFU in the utilization of hypertension and diabetes healthcare services.

The study found various determinants of LTFU behavior among hypertension and diabetes patients, in line with existing literature. For example, higher LTFU rates among less educated individuals are expected, as patients with more education are generally better equipped to understand the importance of adhering to physician-recommended follow-up visits. This finding aligns with studies that underscore education’s positive role in promoting access to and appropriate utilization of healthcare services [16, 22, 23, 25, 26, 28]. Consequently, the establishment of support groups or social media networks (e.g., WhatsApp groups) for hypertension and/or diabetes patients could provide ongoing education on the causes, symptoms, and management of NCDs and their complications. Notably, many patients expressed strong interest in participating in such support groups.

Additionally, the higher likelihood of LTFU among recently diagnosed patients (diagnosed less than two years prior to the interview) may be attributed to a lack of urgency in the absence of severe symptoms, initial denial of the condition, and concerns about adverse side effects of medications [15, 44, 45]. Atinga et al. [21] report that hypertension and diabetes patients in self-denial often reject allopathic medication in favor of herbal and spiritual treatments. This behavior is especially common among newly diagnosed patients, who may take time to accept their condition [46, 47]. Furthermore, patients with chronic diseases like hypertension often become non-compliant when they do not experience significant symptoms, even if they are not strictly following their prescribed medication regimen, and this is often the case for newly diagnosed patients.

The study also revealed that patients in the more urbanized and cosmopolitan Greater Accra Region were more likely to experience LTFU than their counterparts in the Ashanti region. This can be explained by the pressures of busy work schedules, transportation costs, and time lost due to heavy traffic and long waiting times at health facilities [20, 46]. Sani et al. [48] suggest that urbanization is a major factor contributing to the rising prevalence of hypertension in West Africa. In the Greater Accra Region, factors such as busy work schedules, traffic congestion, and the high cost of healthcare are likely to increase the likelihood of LTFU. Appiah et al. [49] similarly found that urban women had higher odds of developing hypertension, though this finding was not significant in their adjusted model.

Another interesting finding of this study is that patients who visited health facilities that provided education on hypertension and diabetes were less likely to experience LTFU. This finding is consistent with the existing literature, which suggests that healthcare facilities that make a conscious effort to educate patients about disease conditions, preventive care, and treatment encourage further visits to those facilities and other healthcare facilities [20, 26]. Similarly, respondents whose treatment conditions were not under control were less likely to experience LTFU, as they required continuous healthcare, a finding corroborated by previous studies [16, 23, 25]. However, some studies have reported contrary results, suggesting that patients with controlled conditions are more likely to experience LTFU because they tend to seek care only when their conditions worsen due to non-adherence to medication [14, 22, 23]. This highlights the need for continuous patient education on the chronic nature of these conditions, particularly for hypertension and diabetes patients, and the importance of adhering to prescribed medication regimens. Changes to medication dosage or cessation of medication should only occur under the guidance of healthcare professionals.

The availability of appropriate healthcare facilities and diagnostic equipment is critical for the effective treatment and management of NCDs. Facilities equipped with the necessary medical diagnostic tools and staffed with physicians who are always available—acting as a “one-stop shop” for patients—help reduce the number of referrals to other facilities and increase efficiency, leading to lower LTFU rates, as noted in prior studies [22, 45]. Even for patients whose healthcare costs are covered by third-party payers, the availability of essential diagnostic equipment reduces both the monetary and time costs associated with obtaining diagnostic tests at other facilities or private laboratories. Therefore, NHIS-credentialed health facilities in Ghana must ensure the availability of essential diagnostic tools and equipment to improve NCD care. Efforts should also be made to significantly increase the supply of physicians, potentially through the recruitment of Ghanaian expatriate physicians, foreign-trained Ghanaian doctors, and medical volunteers. It is equally crucial to eliminate the direct and indirect cost barriers that hinder follow-up visits, as this is vital for the effective management of hypertension and diabetes in Ghana. This requires targeted, comprehensive insurance coverage that pays for all core laboratory tests and medications, particularly for patients over 60 years of age. Additionally, digitalization efforts to support telemedicine and repeat prescription systems are recommended to ease access to care.

Conversely, respondents receiving non-orthodox treatments, such as traditional (herbal/spiritual) remedies or personal/home-made treatments, were less likely to attend follow-up visits at NHIS service providers, especially when they experienced poor treatment outcomes or a lack of improvement with orthodox care [19, 22]. This reinforces findings from earlier studies [20]. Additionally, disrespectful treatment by healthcare workers can foster LTFU, as patients may feel uncomfortable accessing orthodox healthcare services, as reported by [24, 25].

Finally, financial constraints were identified as a major driver of LTFU among hypertensive and diabetic patients, with LTFU being more prevalent among diabetic patients than hypertensive patients. This may be due to the higher costs and lack of NHIS coverage for diabetes diagnostic tests, compared to the lower costs and full NHIS coverage for hypetension diagnostic tests. These co-payment issues, particularly for diagnostic tests and medications, are consistent with earlier findings [14, 26, 46, 47]. Laar et al. [5] observed that difficulties in accessing orthodox medication contribute to non-adherence to hypertension and diabetes treatment, leading patients to seek alternative therapies such as herbal remedies and spiritual healing, which are perceived to be more affordable and effective. The lack of financial resources to manage NCDs has been further confirmed by Atinga et al. [21], who concluded that the high cost of hypertension medications is a major cause of non-compliance. Considering the growing use of herbal medicines in Ghana, especially in rural areas where they are seen as more affordable and effective, there have been calls to regulate the traditional medicine industry to ensure the safety of its users. This study suggests that integrating traditional herbal medicine into the mainstream healthcare system may enhance adherence to non-communicable disease (NCD) treatment and management. Collaboration between traditional healers and orthodox healthcare providers may help reduce LTFU and foster greater trust in the healthcare system.

## Conclusions

This study has demonstrated that non-adherence to physician-recommended follow-up visits (LTFU) among hypertension and diabetes patients is pervasive in Ghana, with slightly more than a quarter (27%) of patients being lost to follow-up. If not addressed, this phenomenon could lead to an increase in preventable deaths and the worsening of disease symptoms. A range of household and patient characteristics, provider and treatment factors, as well as financial considerations were found to influence LTFU behavior. Key predictors of LTFU behavior include region of residence, the number of years since diagnosis, awareness of the need for follow-up visits, the availability of physicians and diagnostic equipment at healthcare facilities, and patient stigmatization, among others.

While the study’s findings are generally valid, there are few limitations worth acknowledging. First, the analysis focused only on patients recorded in the NHIA electronic claims database from 2019 to 2020, rather than the majority of claims processed manually, as only 9% of service providers submitted electronic claims at the time the data was collected. Second, the study's geographical scope was limited, with respondents selected from only two of Ghana’s 16 administrative regions. Third, the study focused solely on patients diagnosed with hypertension and diabetes, excluding those with other NCDs or infectious diseases. Lastly, the number of respondents in the control group was relatively small. Despite these limitations, the findings align with those reported in prior literature.

## Supplementary Information

Supplementary Material 1.

Supplementary Material 2.

## Data Availability

The datasets generated and/or analysed during the current study are available upon reasonable request from the corresponding author at enamponsah@ug.edu.gh. Request for the data can also be channeled to PharmAccess Foundation, Ghana at g.sunkwamills@pharmaccess.org.

## Abbreviations

DM: Diabetes milletus
GDP: Gross domestic product
HPT: Hypertension
LFFU: Lost-to-follow up
NCDs: Non-communicable diseases
NHIA: National Health Insurance Authority
NHIS: National Health Insurance Scheme
SDGs: Sustainable Development Goals

## Footnotes

1 The sample size is determined by the equation:

$$n=[\text{DEFF}*\text{Np}(1-p)]/ [({d}^{2}/{{Z}^{2}}_{1-\alpha /2}*(N-1)+p*(1-p)]$$

where: *n* = sample size. DEFF = design effect (used in cluster surveys). *N *= population size. *p* = the hypothesized % frequency of outcome factor in the population. *q* = 1-*p*. *d* = confidence limits. See: http://www.openepi.com/SampleSize/SSPropor.htm.

2 The sample size is determined by the equation:

$$n=[\text{DEFF}*\text{Np}(1-p)]/ [({d}^{2}/{{Z}^{2}}_{1-\alpha /2}*(N-1)+p*(1-p)]$$

where: *n* = sample size. DEFF = design effect (used in cluster surveys). N = population size. *p* = the hypothesized % frequency of outcome factor in the population. *q* = 1-*p*. *d* = confidence limits. See: http://www.openepi.com/SampleSize/SSPropor.htm.

3 In this section, all such counterintuitive results will not be discussed. We believe this is a methodological limitation of the study, caused by having a relatively smaller proportion of the respondents sampled from the control group (27%) than the case group (73%). See Table 1. Interpretation of the results should be done with this limitation in mind. It is equally important to note that the control group constitutes slightly more than a quarter (27%) of the sample.

4 In this section, all such counterintuitive results will not be discussed. We believe this is a methodological limitation of the study, caused by having a relatively smaller proportion of the respondents sampled from the control group (27%) relative to the case group (73%). See Table 1. Interpretation of the results should be done with this limitation in mind. It is equally important to note that the control group constitutes slightly more than a quarter (27%) of the sample.
