# Supplementary material for: Determinants of lost-to-follow-up (LTFU) among National Health Insurance Scheme-insured hypertension and diabetes patients attending accredited health facilities in Ghana
Source: Trop Med Health. 2025 May 6;53:65. doi: 10.1186/s41182-025-00743-3 (PMC12054282; doi:10.1186/s41182-025-00743-3)
Supplement: Supplementary file 1 — Supplementary Material 1. [file 41182_2025_743_MOESM1_ESM.zip › Appendix 1/Appendix Table 1.2.docx]

Appendix Table 1.2

| Variable | Definition/Measurement |
| --- | --- |
| Outcome variable |  |
| LFFU | 1 if patient made only one visit in 2019 for hypertension and diabetes treatment and management and no further visit in 2020; otherwise 0 |
|  |  |
| *predictor variables* |  |
| Sex | 1 if patient is female; otherwise, 0 |
| Region | 1 if patient is resident in Ashanti region (ref. Greater Accra Region) otherwise, 0 |
| **Age** |  |
| Adults (25-64years) | 1if patient is in the age range 25-64 years (reference) |
| Seniors (≥65 years) | 1 if patient is aged 65 years or more; otherwise, 0 |
| **Household size** |  |
| Small size (≤3) | 1 if household has three or fewer members (reference) otherwise 0 |
| Medium-size (4-6) | 1 if household has membership of 4 to 6 members; otherwise 0 |
| Large size (≥7) | 1 if household has seven or more members; otherwise, 0 |
| **Educational Level** |  |
| ≤Primary | 1 if patient has primary or less education (reference) |
| ≥Secondary | 1 if patient has secondary education or better; otherwise, 0 |
| **Ethnicity** |  |
| Akan | 1 if the patient belongs to the Akan ethnic group; otherwise, 0(reference) |
| Non-Akan | 1 if the patient belongs to an ethnic group other than Akan; otherwise, 0. |
| **Marital Status** |  |
| Single | 1 if patient is single or unmarried; otherwise, 0 (reference) |
| Married | 1 if patient is married otherwise 0 |
| **Patient’s HPT/DM Status** |  |
| HPT & DM | 1 if patient has both diabetes and hypertension; otherwise, 0 (reference) |
| HPT | 1 if patient has hypertension only; otherwise, 0 |
| DM | 1 if patient has diabetes only; otherwise, 0 |
| **First Diagnostic period** |  |
| Diagnostic period (≤2 years) | 1 if patient was first diagnosed of HPT or DM two or less years ago at the time of the survey in February/March 2022; otherwise, 0 |
| Diagnostic period (5-10 years) | 1 if patient was first diagnosed of HPT or DM 5-10 years ago at the time of the survey in February/March 2022; otherwise, 0 |
| Diagnostic period (>10 years) | 1 if patient was first diagnosed of HPT or DM 10 or more years ago at the time of the survey in February/March 2022; otherwise, 0 |
| Follow-up awareness | 1 if patient is aware of the need to make scheduled visit to the health facility due to the chronic nature of the condition, otherwise 0 |
| Patient currently receiving treatment | 1 if patient is currently receiving treatment; otherwise, 0 |
| Family member with NCD | 1 if any family member has an NCD condition otherwise, 0 |
| HPT/DM Support Group |  |
| Strongly recommend | 1if the patient strongly recommends the establishment of an HPT/DM support group at the health facility otherwise 0 |
| Neutral | 1 if patient is neutral about recommending the establishment of an HPT/DM group at the health facility. |
| Education on HPT/DM diagnostic procedure | 1 if patient receives education on diagnostic procedure at the health facility. |
| **Education on condition** |  |
| All education | 1 if patient receives complete education from health facility on HPT and DM (reference); otherwise, 0 |
| Symptoms and Control | 1 if patient receives education from health facility on symptoms and control of HPT and DM; otherwise, 0 |
| Causes and control | 1 if patient receives education from health facility on causes and control of HPT and DM; otherwise, 0 |
| **Availability of physicians** |  |
| Available all the time | 1 if physician is available in the facility all the time (reference); otherwise, 0 |
| Available most of the time | 1 if physician is available in the facility most of the time otherwise 0 |
| Available some of the time | 1 if physician is available in the facility some of the time otherwise 0 |
| **Availability of HPT/ DM Medication** |  |
| Always Available | 1if medication for HPT/DM is available in the health facility all the time (reference) otherwise 0 |
| Available most of the time | 1 if medication for HPT/DM is available in the health facility most of the time; otherwise, 0 |
| Available some of the time | 1 if medication for HPT/DM is available in the health facility some of the time; otherwise, 0 |
| Unavailable always/most of the time | 1 if medication for HPT/DM is unavailable in the health facility always/most of the time otherwise 0 |
|  |  |
| **stigmatization** | 1 if patient responded that she felt or perceived stigmatized (discriminated against, insulted, disrespected) by healthcare workers at the facility due to her condition otherwise 0 |
|  |  |
| **Patients’ current treatment** |  |
| Orthodox only | 1 if patient utilizes only allopathic or modern medicine otherwise, 0 (reference) |
| Non-orthodox only | 1 if patient utilizes only non-orthodox medicine (herbal medicine and other home-made remedies) for NCD (hypertension and diabetes); otherwise, 0 |
|  |  |
| NHIS HPT/DM tests coverage | 1 if patient responded that the insurance coverage for HPT/DM tests is inadequate otherwise 0 (adequate) |
| **Patient’s annual income** |  |
| Below median income (GHc5,792)  Above median income (GHC5,792) | 1If patient’s annual income is below the median income (reference) otherwise 0  1 if patient’s annual income is above the median income; otherwise, 0 |
| Patient’s out-of-pocket payment | 1 if patient makes out-of-pocket payments for HPT and DM healthcare; otherwise, 0 |
|  |  |
